# Inhibition of Urease by Disulfiram, an FDA-Approved Thiol Reagent Used in Humans

**DOI:** 10.3390/molecules21121628

**Published:** 2016-11-26

**Authors:** Ángel Gabriel Díaz-Sánchez, Emilio Alvarez-Parrilla, Alejandro Martínez-Martínez, Luis Aguirre-Reyes, Jesica Aline Orozpe-Olvera, Miguel Armando Ramos-Soto, José Alberto Núñez-Gastélum, Bonifacio Alvarado-Tenorio, Laura Alejandra de la Rosa

**Affiliations:** Departamento de Ciencias Químico-Biológicas, Instituto de Ciencias Biomédicas, Universidad Autónoma de Ciudad Juárez, Ciudad Juárez, Chihuahua 32310, Mexico; ealvarez@uacj.mx (E.A.-P.); alejandro.martinez@uacj.mx (A.M.-M.); laraguirre30@gmail.com (L.A.-R.); al113457@alumnos.uacj.mx (J.A.O.-O.); al113429@alumnos.uacj.mx (M.A.R.-S.); jose.nunez@uacj.mx (J.A.N.-G.); bonifacio.alvarado@uacj.mx (B.A.-T.); ldelaros@uacj.mx (L.A.R.)

**Keywords:** urease, inhibition, disulfiram

## Abstract

Urease is a nickel-dependent amidohydrolase that catalyses the decomposition of urea into carbamate and ammonia, a reaction that constitutes an important source of nitrogen for bacteria, fungi and plants. It is recognized as a potential antimicrobial target with an impact on medicine, agriculture, and the environment. The list of possible urease inhibitors is continuously increasing, with a special interest in those that interact with and block the flexible active site flap. We show that disulfiram inhibits urease in *Citrullus vulgaris* (CVU), following a non-competitive mechanism, and may be one of this kind of inhibitors. Disulfiram is a well-known thiol reagent that has been approved by the FDA for treatment of chronic alcoholism. We also found that other thiol reactive compounds (l-captopril and Bithionol) and quercetin inhibits CVU. These inhibitors protect the enzyme against its full inactivation by the thiol-specific reagent Aldrithiol (2,2′-dipyridyl disulphide, DPS), suggesting that the three drugs bind to the same subsite. Enzyme kinetics, competing inhibition experiments, auto-fluorescence binding experiments, and docking suggest that the disulfiram reactive site is Cys592, which has been proposed as a “hinge” located in the flexible active site flap. This study presents the basis for the use of disulfiram as one potential inhibitor to control urease activity.

## 1. Introduction

Urease activity (E.C. 3.5.1.5) constitutes one of the biological steps in the global nitrogen cycle [1,2]. It is present in bacteria, fungi and plants and is one of the enzymes selected as a target for controlling medical [3], agricultural [4] and environmental issues [5]. Structural and mechanistic features of urease are largely addressed in the literature [6,7,8,9,10,11,12,13,14,15,16,17,18,19]. It is accepted that the active site architecture and mechanism of action are similar for all ureases, independent of the extraction source [7,13,16,18,20]. Urease is an amidohydrolase that rapidly produces the decomposition of urea into ammonia and carbamate, followed by the spontaneous decomposition of carbamate into bicarbonate and a second ammonia molecule. The reaction involves the participation of two catalytic Ni^2+^ ions bound to a carbamylated Lys that fulfills the structural function of the active site [6,7,10,11,16,21] but does not participate directly in the catalysis and an important His residue functioning as the general acid catalyst [7]. The Ni centers and the carbamylated Lys residue are located in the active site and are relatively immobile, but His593 (Jack bean urease, JBU numeration, here after used) is located in a flexible flap that opens and closes [10] during the catalytic cycle. Therefore this acid-acting residue is not always in the proton transfer subsite, and seems to be required only inside the active site of the productive complex to assist in catalysis once the flap is in the closed conformation [18,22,23]. It has been shown that blocking the closure of active site flap produces a significant inhibition of urease activity [10,12,24,25], most likely by obstructing the correct accommodation of catalytic His593. Thus, one of the strategies to control urease activity is the use of compounds that block the freedom of the flexible flap. Inhibition studies together with site-directed mutagenesis showed the role of Cys592 as a flap hinge in the observed flexibility [12,25,26]. Mutation of the Cys592 to Ala showed that the *Helicobacter pylori* mutant enzyme was less susceptible to inhibition by epigallocatechin and quercetin [25], supporting the proposed role for this residue. The description of compounds interacting and blocking Cys residues in urease enzymes, especially residue 592, is extensive [9,24,25,27,28,29,30,31,32,33]. These compounds include those that contain groups that are reactive to thiols. It is suggested that the mechanism of action of such compounds is their interaction with Cys592. Here we used the urease from seeds of *Citrullus vulgaris* (CVU), a plant enzyme, to demonstrate that disulfiram (DSF)—a reactive sulphur-containing compound that is approved by the FDA for clinical use in humans—is a potential effective urease inhibitor. We also used four compounds that contain groups that are reactive to thiols to describe the potential interaction of DSF with Cys592 by means of kinetic and molecular docking experiments. Molecular docking plays an important role in the rational design of drugs, being a useful tool which reasonably predicts the best orientation of one molecule within the putative target, allowing the performance of reliable virtual screening processes; for instance, see [34,35,36]. Here the docking approach seems to be adequate to predict if compounds of this kind can interact with relevant Cys residues.

## 2. Results

### 2.1. Kinetic Characterization of Urease from C. vulgaris Seeds

**Figure 1:**
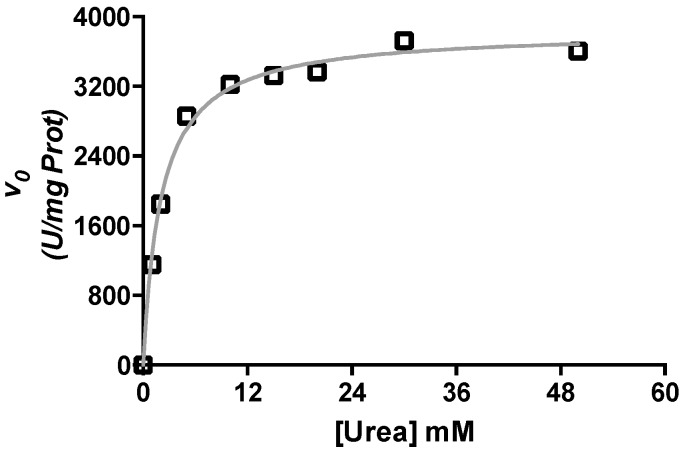
Saturation kinetics of *Citrullus vulgaris* urease (CVU) by urea. The observed initial velocities of reaction are plotted against diferent concentrations of urea. U is defined as the amount of enzyme that produces one micromol of NH_3_^+^ min^−1^·mL^−1^. The grey line shows the best curve fitting to Equation (1). Non-linear curve fitting and plot were prepared using Graph Pad Prism 5^®^ (GraphPad Software, Inc., La Jolla, CA, USA) and one of three typical experimental results is used here as indicated in the Methods section.

CVU kinetic parameters on the reaction of urea hydrolysis were determined. We observed a *V*_max_ of 3841.00 ± 67.15 U/mg of protein and a *K*_m_ of 2.08 ± 0.18 mM at pH of 6.80 (Figure 1). These values were compared with those reported elsewhere in the literature [37] and found to be in agreement with previous work done at pH of 8.00, which are: *V*_max_ of 3700 U/mg of protein and a *K*_m_ value of 8 mM. Given the observed results we assumed that our CVU preparation was suitable to perform the inhibition studies.

### 2.2. Kinetic Characterization of DSF Inhibition over Citrullus vulgaris Urease

**Figure 2:**
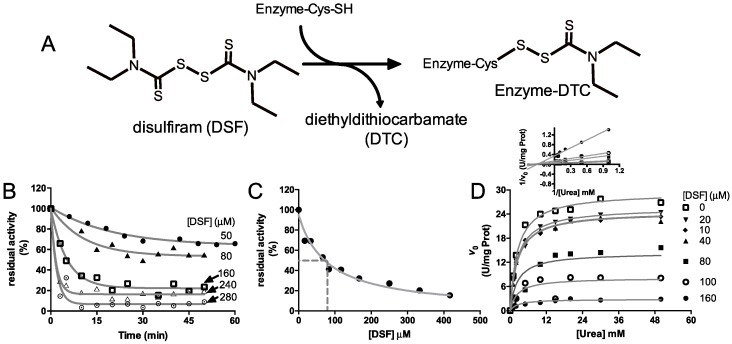
Kinetic characterization of the inhibition of CVU by disulfiram. (**A**) DSF general reaction of solvent-accessible Cys residues in enzymes. Susceptible Cys residues are carbamylated with a diethylthiocarbamate moiety (DTC) followed by the release of a proton together with the free DTC moiety. (**B**) Time courses of inactivation of CVU pre-incubed with different DSF concentrations at 37 °C. Solid grey lines show the best curve fitting to a single exponential decay equation. Each curve is labelled with the DSF concentration used. (**C**) Inhibition kinetics of urease observed by the incubation with DSF. The solid grey line shows the best curve fitting to Equation (3). The dashed grey line shows the concentration of DSF at 50% of total inhibition. (**D**) Inhibition kinetic pattern obtained by measuring the saturation kinetics of CVU by urea at variable/fixed concentrations of DSF. The solid grey line shows the best curve fitting to Equation (5). The insert in D shows the Lineweaver–Burk plots of the kinetic pattern where intersection of linear curves to abscise axis is observed, consistent with a non-competitive inhibition. Here U is defined as the amount of enzyme that produces the change of one unit of the absorbance at 558 nm per milligram of protein. Non-linear and linear curve fitting and plots were prepared using Graph Pad Prism 5^®^. The presented experiments are one of three typical results. Chemical structures were drawn using ACD/ChemSketch^®^ (Advanced Chemistry Development, Inc., Toronto, ON, Canada).

It is known that DSF reacts with solvent-exposed Cys residues in some enzymes [38,39], forming a covalent adduct and leaving a diethylthiocarbamate moiety (DTC) in the protein (Figure 2A). If this modification occurs in residues that account for activity, an inhibition is expected. To explore the susceptibility of CVU to inhibition by DSF, the urease activity was monitored in the presence of different concentrations of compound, over different times of incubation at 37 °C. DSF produces a time-dependent progressive loss of urease activity that follows a pseudo-first order kinetics, that reach a *plateau* and depend on inhibitor concentration (Figure 2B). The observed remaining enzyme activity at *plateau* apparently corresponds to the uninhibited enzyme fraction at equilibrium. Each data set was fitted to Equation (2). The observed inactivation kinetic constant increases in a non-linear trend as a function of DSF concentration used in the experiment, consistent with an inactivation mechanism consisting of two or more steps. One of these steps may be the binding of DSF and at least the second could be accounting to the reaction of one of the enzyme Cys residue with the inhibitor and subsequent formation of the DTC derivative.

In these conditions it was observed that 50 minutes of incubation with DSF is enough time to assure that apparent equilibrium is reached at any used concentration, i.e., in incubation of CVU with 50 µM an apparent half-life time of 13.80 min, a *k*_obs_ of 0.050 ± 0.007 min^−1^ and a *plateau* at 63.60% ± 1.40% of the initial velocity were observed and after 50 min the *plateau* was reached. For 80 µM, an apparent half-life time of 7.50 min, a *k*_obs_ of 0.092 ± 0.024 min^−1^ and a *plateau* at 53.48% ± 3.05% of the initial velocity were observed around 25 min after addition of DSF.

In order to estimate the apparent inhibition constant (*IC*_50_), CVU was incubated for an hour at 37 °C with different concentrations of DSF and the remaining initial velocities of enzyme were then measured (Figure 2C). The inhibition followed a hyperbolic trend with an *IC*_50_ value of 80.02 ± 1.30 × 10^−4^ μM, obtained by fitting data to Equation (3). This apparent *K*_i_ value was used to design an inhibition pattern experiment (Figure 2D) to better explain the mechanism of DSF inhibition. Lineweaver–Burk plots of the inhibition pattern indicated a non-competitive inhibition mechanism (Figure 2D insert); thus, initial velocity data in Figure 2D were globally fitted to Equation (5). The obtained inhibition constant (*K*_i_) was 67.60 ± 7.00 μM, which is comparable to *IC*_50_ value. The observed non-competitive inhibition could be explained by the depletion of active CVU forms, i.e., DSF reacts with Cys592 forming an enzyme-inhibitor complex, we hypothesized that the active site flap in enzyme-DSF complex is unable to completely close, a position that is essential for the correct accommodation of the catalytic His593.

### 2.3. Inhibition of C. vulgaris Urease by Other Thiol Reactive Compounds

**Figure 3:**
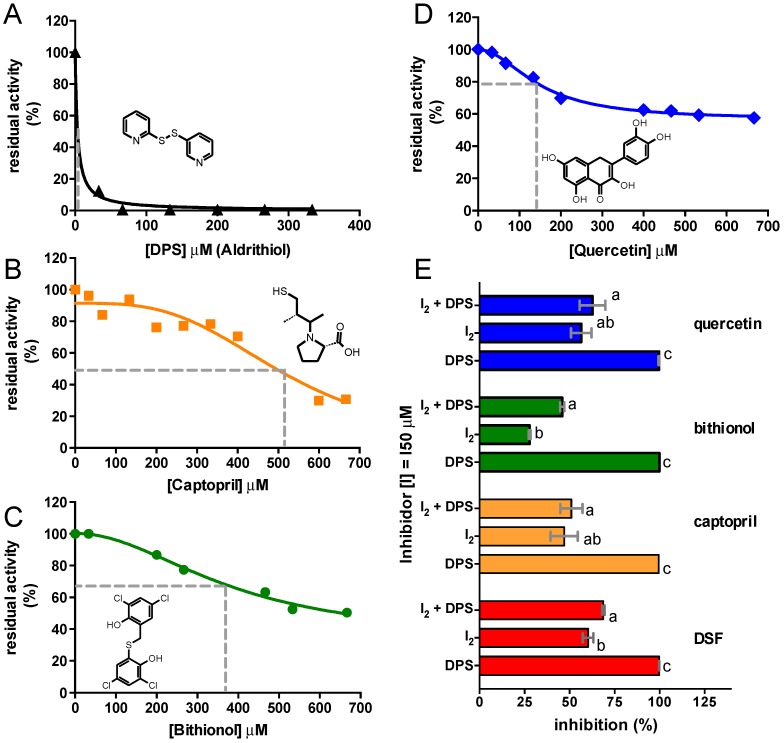
Inhibition of CVU with a specific thiol-reagent and other reactive drugs. (**A**) Inhibition kinetics of CVU by DPS; (**B**) Captopril; (**C**) Bithionol and (**D**) Quercetin. The solid lines in A and B show the best curve fitting to Equation (3), and the solid lines in C and D show the best curve fitting to Equation (4); the latter corresponds to a partial inhibition equation, where the initial and equilibrium residual activity (span) is considered for the *IC*_50_ calculation. The chemical structures of inhibitors are depicted in the corresponding graph and were drawn using ACD/ChemSketch^®^. The inhibition curve produced by DSF is depicted in Figure 2C. The dashed grey line shows the concentration of inhibitor at 50% of total inhibition (**E**) Percentage of inhibition produced by drugs (I_2_: captopril, bithionol and quercetin) and DPS at the concentration equal to the *IC*_50_ value. In I_2_ + DPS, CVU was incubated with a given drug for 1 h and then DPS was added and immediately the residual activity was measured, showing that I_2_ protects CVU from full DPS inhibition. The experiment was reproduced three times, and mean and standard error are plotted. A one-way ANOVA was performed to find significant differences between treatments. The different characters depicted on bars indicate significant differences at *p* < 0.05. Plots were prepared using Graph Pad Prism 5^®^.

In order to investigate the susceptibility of CVU to inhibition by other known thiol reagents, and associate DSF inhibition of CVU to its possible interaction with a Cys residue, the effect of the thiol reagent DPS and other drugs that possess groups that react with thiols were tested: captopril, bithionol and quercetin-these compounds inhibit urease activity in a time dependent trend in a similar manner than for DSF. The *IC*_50_ values between these compounds were of the same order, except for DPS, which was near the submicromolar range (Figure 3 and Table 1).

It was demonstrated that the reactivity and titration of essential thiol of urease from *Klebsiella aerogenes* (KAU) is affected by the presence of substrate and competitive inhibitors [40]. It was also demonstrated that pre-incubation of KAU with the active site competitive inhibitor phenyl-phosphorodiamidate (PPD), protects one Cys residue per catalytic unit from DPS modification during thiol group titration experiments, showing a possible interaction between PPD, or DPS and the active site Cys, most likely with Cys592. It has also been previously reported that quercetin exerts inhibition of KBU by its interaction with Cys592 [25]. Given these findings, it is assumed that, DPS reacts in specific manner with Cys residues, so the complete inhibition observed in CVU is produced by its interaction with essential Cys residues. It was also surmised that enzyme activity protection against DPS elicit by the pre-incubation of CVU with less reactive inhibitors could be used as a tool to detect compounds that interact with relevant Cys residues. Whether captopril, bithionol, and quercetin (here called I_2_) compete for at least one same site as DPS was investigated by measuring their protective effect over DPS full inhibition (Figure 3E). After the incubation of CVU with I_2_ for one hour, DPS was then added, and the residual activity was measured. It was found that pre-incubation of enzyme with thiol reactive compounds protected urease from the full inhibition elicited by DPS. Our results suggest that the inhibition produced by DSF is most likely due to the blocking of Cys592. The competition experiments between PPD and DPS [40] and with quercetin [25] performed in KBU also support this suggestion.

### 2.4. Binding of DSF to Citrullus vulgaris Urease Revealed by Fluorescence Auenching Experiments

**Figure 4:**
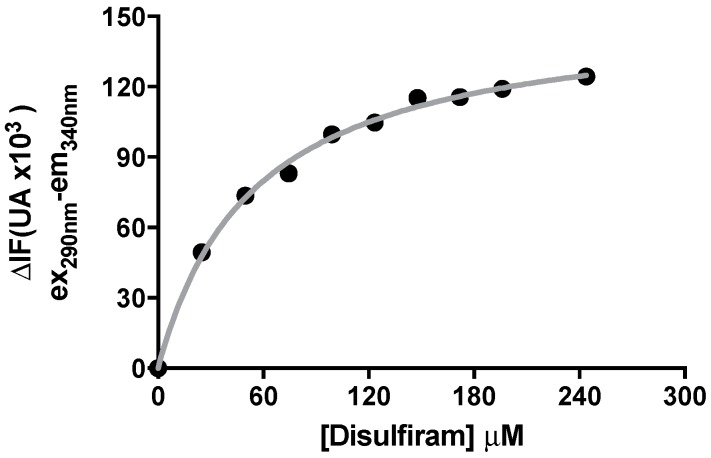
Binding of DSF to *C. vulgaris* urease at equilibrium. The change in the intrinsic fluorescence (ΔIF) was measured an hour after the addition of the indicated concentrations of DSF. A non-linear curve fit to Equation (5) was made and is indicated by the solid grey line. Curve data is obtained in one of three identical binding saturation experiments. Non-linear curve fitting and plots were prepared using Graph Pad Prism 5^®^.

The apparent dissociation constant of DSF from CVU was determined by measuring protein fluorescence quenching. We found that incubation of enzyme with DSF produces a quenching in CVU autofluorescence (Figure 4). The change in fluorescence quenching of CVU exercised upon the addition of DSF to CVU was in a hyperbolic trend, thus fluorescence data was fitted to Equation (6). The resulting DSF dissociation constant (*K*_d_ = 54.90 ± 3.90 μM) is comparable to that of *IC*_50_ and *K*_i_ constants. The change in autofluorescence produced by DSF was also time-dependent, and, therefore, all measurements were made an hour after addition of the drug. This time was sufficient to assure that the maximum changes were reached.

### 2.5. Binding of DSF to Citrullus vulgaris Urease Revealed by Docking Experiments

**Figure 5:**
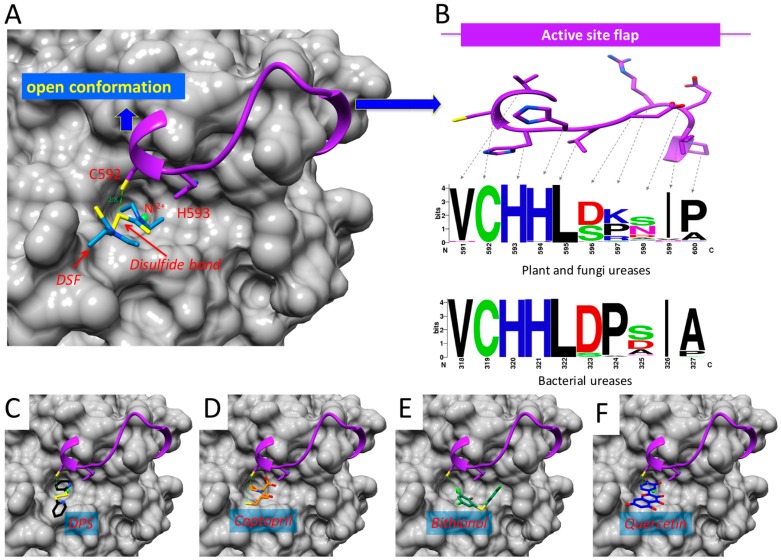
Binding model of DSF and other reactive compounds to urease. (**A**) Model of the binding of DSF to JBU enzyme showing the interaction with Cys592 (dashed line, distance in Å). Ni^2+^ ions are shown in ball representations, active site flexible flap is shown in cartoon representations with Cys592 and His593 depicted in stick models and the rest of JBU is in surface representation. (**B**) Frequency of residues present in the active site flexible flap. The multiple alignment of urease enzymes available in Data Bank was used to plot frequencies in the logo. The amino acid residues are depicted as color code on the basis of their physicochemical properties as stated default it the weblogo server (http://weblogo.berkeley.edu/logo.cgi: polar amino acids (G,S,T,Y,C,Q,N) are green, basic (K,R,H) blue, acidic (D,E) red and hydrophobic (A,V,L,I,P,W,F,M) are black). (**C**) Model of the binding of DPS (**D**) Captopril; (**E**) Bithionol and (**F**) Quercetin to JBU. All structural images were generated using USCF-Chimera and are represented in a similar way to A.

Since the genetic information of CVU is not yet available, the possibility of DSF of interacting with active site flap Cys592 was investigated by docking approach and by a comparison of the residues present in the flap of all ureases homologs found in the NCBI-non-redundant protein library. For the docking modelling, the crystallographic structure of JBU (PDB code 4AL3) and the three-dimensional model of DSF (ChemSpider ID: 3005) were used. The mechanistic and structural aspects of urease enzymes are well documented, and it is accepted that ureases work within a general mechanism regardless of whether they come from a plant, fungi or bacteria. Here it is anticipated that docking results of DSF in JBU could give a good idea of what is happening in CVU and also what would happen in other ureases. Our docking results show that DSF binds to the active site of JBU in the open flap conformation where a direct interaction between one of the disulfur of the tetrathiuram and the sulfur atom of Cys592 is observed (Figure 5A). Also, docking binding DPS experiments (ChemSpider ID: 58603), Captopril (ChemSpider ID: 40130), Bithionol (ChemSpider ID: 2313) and Quercetin (ChemSpider ID: 4444051) were performed (Figure 5C–F). All compounds interact with active site flap, an observation that supports the suggestions made on the basis of the inhibition experiments. The presence of two neighboring His residues around Cys592 is suggestive of the origin of the observed high thiol reactivity. It is well known that this type of residue causes a decrease in the intrinsic pK_a_ of this group. For the possibility of DSF to inhibit any known urease, an analysis of the evolutionary conservation of residues located in the active site flap of urease enzymes was performed. Based on the premise that the motive Cys592-His593-His594 accounts for the inhibition exerted by compounds that contain sulfur-reactive groups, it is proposed that any urease containing at least these three residues would be inhibited by this type of compounds. A multiple alignment with a 1000 non-redundant bacterial sequences as well as another multiple alignment with 1035 non-redundant plant and fungi sequences were performed. The region corresponding to the flexible flap in all bacteria, plants and fungi were plotted in a webLogo graph (Figure 5B). A conserved domain for all ureases VCHHL was observed that corresponds to position 591 to 595 in plant ureases and 318 to 322 in bacterial ureases. The fact that VCHHL is a conserved cluster from bacteria trough fungi and plants suggests that these residues play an important and universal role in the function of urease enzymes. Meanwhile, residues of the C-terminal region are variable. Since it appears that in all urease flexible flaps, the CHH is present, seems that any compound containing reactive sulphur groups can inhibit any urease. Considering the biochemical characterization, the docking and the sequence conservation results, it is suggested that DSF is a universal urease inhibitor.

## 3. Discussion

### 3.1. Potential Use of Disulfiram to Control Urease Activity

Although inhibition of urease enzyme has been widely described, new urease inhibitors are still being sought. Inhibition by natural products isolated from plants as well as other synthetic designed compounds [24,30,31,41,42,43,44,45,46,47,48,49] showed in most cases competitive or non-competitive inhibition mechanisms with *IC*_50_ ranging from 2 to 300 μM. In many instances, inhibition has been attributed to the interaction of compounds with a Cys residue in the hinge flap [8,9,29,30,31,32]. In *Kleibsiella aerogenes* urease, substitution of Cys for Ala in the flexible loop produced an enzyme that shifted from being inhibited by epigallocatechin and quercetin, to a barely inhibited form [25], suggesting that inhibition was produced by the interaction of this compound with the Cys in the flexible loop. Our results are consistent with reports describing the interaction of inhibitors with Cys592 [25,26,40]. The observed value of DSF *K*_i_ is in the range of other compounds proposed as potential inhibitors to control the activity of urease enzymes.

Finding new applications for a known drug constitutes one of the strategies for discovering new antimicrobial agents. Here the inhibition produced by DSF over CVU is presented. It was shown that DSF inhibits the mitochondrial aldehyde dehydrogenase 2, through the modification of the essential Cys302 [50,51]. This is how the drug exerts its effect over ethanol metabolism in patients with chronic alcoholism. Although it is known that the thiocarbamate group of DSF interacts with multiple targets, and doses of 500 mg have been accepted in humans even with their side effects [52]. In this context, it was recently shown that DSF inhibits the carbamate kinase of *Giardia lamblia* and kills their trophozoites. The basis of these inhibitions was recently described in terms of structural crystallographic data [39]. This is produced by modification of a Cys residue located at the edge of the active site, preventing during the reaction an important conformational transition of a loop adjacent to the ADP/ATP binding site. DSF also inhibits betaine aldehyde dehydrogenase from *Amaranthus hypochondriacus* and *Pseudomonas aeruginosa* [51,53], and it was also suggested that DSF may be used to inhibit the growth of *P. aeruginosa* during infections [38,51]. Structural studies showed that the inhibition of these dehydrogenases is produced by modifying the catalytic active site Cys residue [38,51]. It has also been demonstrated that DSF and copper ions can kill *Mycobacterium* *tuberculosis* synergistically [54]. It was also shown that DSF could inhibit HCV replication to a similar extent as the clinically used antiviral agent [55]. In this study it is proposed that observed DSF urease inhibition involves the formation of a disulphide bond between DSF and the hinge Cys of the flexible flap and blocks its closure. Briefly, the proposed and accepted catalytic mechanism of urease [15,18,19] consists in the following (Figure 5): (1) CVU may be in equilibrium in the closed and open conformations; (2) urea substrate binds to the enzyme in the open conformation; (3) this is followed by a change to the closed conformation, in which His593 approaches the active site and functions as the general acid needed for catalysis [7]; (4) urea decomposition to NH_4_^+^ and carbamate ion (NH_2_COO^−^) takes place in the active site; (5) finally, the products are released of urease in the open conformation. Taking this general mechanism into account it is suggested that DSF reacts with Cys592 producing *N,N*-diethyldithiocarbamate (DTC) that impedes the closure of the flap. This yields a less active urease because the proton transfer steps are compromised.

### 3.2. Proposed Mechanism of Urease Inhibition by Disulfiram

**Figure 6:**
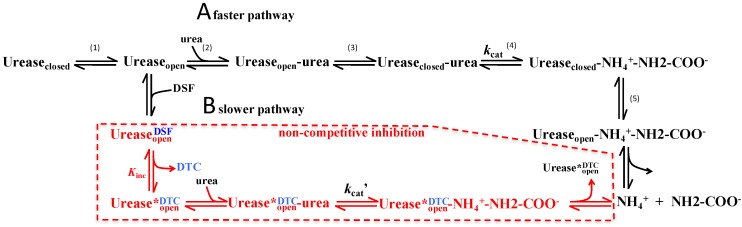
Scheme of the proposed kinetic mechanism for the inhibition of urease enzymes by disulfiram. (**A**) In the absence of inhibitor urea binds to urease in the open conformation; followed by the change to the closed conformation and allowing general base to accommodate in the correct position; subsequently the hydrolysis is produced and the flap changes to the open conformation; allowing the release of products and water/-OH exchange. (**B**) In the presence of DSF, formation of adduct with urease in the open conformation impedes the full closure of the flap and thus DSF derivative obstructing the accommodation of the general acid producing a less active form of urease. Urease*DTC-open is the proposed form of the trapped enzyme as opposed to a not fully-closed or fully-open inhibited conformation.

**Figure 7:**
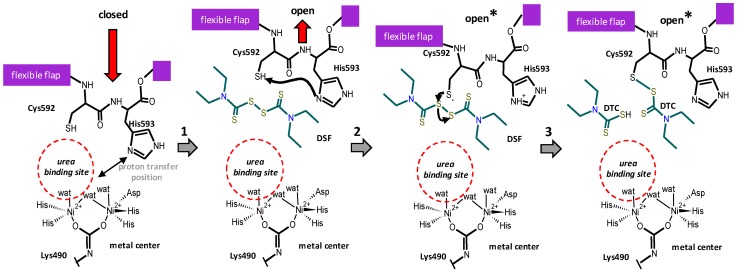
Scheme of the proposed chemical mechanism for the inhibition of urease enzymes by disulfiram: Empty urease is able to be in the closed or open conformation; (**1**) DSF binds to CVU in the open conformation and (**2**) Cys592 thiolate produces the attack to the thiuram group; (**3**) the enzyme is then modified by DTC moiety and block the flap to achieve the closed conformation; finally, the other DTC moiety is released from the enzyme. Chemical structures were drawn using ACD/ChemSketch^®^.

The observed inhibition of CVU by DSF is due to a reaction that produces a thiol–disulfide exchange. In *Giardia lamblia* carbamate kinase DSF reacts with Cys242 and forms a covalent product detected by crystallography and mass spectrometry [39]. The products consist in a DTC moiety as the one showed in Figure 2A. In the case of betaine aldehyde dehydrogenase, a similar thiol–disulfide exchange with the catalytic Cys was also proposed [38,51,53]. Although in urease enzymes Cys591 seems to be highly reactive, the underlying molecular mechanism needs to be addressed. But given the large amount of information about the mechanism of function of urease, we anticipated that Cys592 in the open flap conformation is susceptible to attacking the thiuram group of DSF (Figure 6 and Figure 7). (1) It is suggested that His593 is able to take the proton from thiol group of Cys592 producing the thiolate form, which is more reactive. (2) After this Cys activation, thiolate produces an attack to the thiuram moiety of DSF and generates the Urease-DTC complex. (3) Finally, Cys modification causes interference not only with the closure of the active site flap but blocks general acid His593 to efficiently exchange a proton during catalysis.

## 4. Materials and Methods

### 4.1. Urease Preparation

CVU was purified to apparent homogeneity using DEAE-Sepharose (GE, Healthcare Life Sciences, Pittsburgh, PA, USA) and Q-Sepharose Hi-trap cromatography (GE, Healthcare Life Sciences). Briefly, 100 g of clean and dry seeds were pulverized in a blender at room temperature, and the resulting powder was mixed for four hours (here after sample was handled in cold) with 100 mL of extraction buffer (50 mM Hepes, 50 mM NaCl, at pH of 6.8). Then, the homogenate was centrifugated at 12,000 rpm for an hour and supernatant was recovered and passed through 50 mL DEAE-Sepharose column, then loaded column was washed with 300 mL of extraction buffer and eluted using a linear gradient of NaCl from 50 to 500 mM in extraction buffer using FPLC equipment (Bio-Rad, BioLogic^®^, Hercules, CA, USA). Fractions with urease activity were pooled and dialyzed exhaustively in a membrane tube of 3 kDa (Sigma, Toluca, Estado de México, México) against extraction buffer. The desalted urease, was passed through 5 mL Hi-Trap Q-Sepharose HT column, washed with 300 mL of extraction buffer and eluted with a NaCl linear gradient from 50 to 500 mM in extraction buffer. Finally, the fractions containing urease activity were pooled and used for further analysis.

### 4.2. Enzyme Activity Assay and Kinetic Characterization

The specific ureolytic activity was assayed spectrophotometrically at 37 °C in a Fluostar Omega (BMG LABTECH Inc, Cary, NC, USA) spectrometer. The reaction was monitoring by the increase of ammonia concentration following the absorbance at 558 nm by The Phenol Red Assay, taking similar considerations as the one described elsewhere [15,56]. The standard urease test consisted in 0.3 mL of reaction buffer: 0.5 mM MES, 0.016 mM phenol red, and 50 mM urea, at pH 6.8. All assays were initiated by the addition of the enzyme (≈0.6 ng/mL). Initial velocity rates were determined from the initial linear portion of the reaction course-times. Saturation kinetics experiments were performed under the same conditions, except that different concentrations of urea were used (0.5 to 50 mM), and data were fitted to Michaelis-Menten model (Equation (1)). All experiments presented were reproduced at least three times.

Equation (1): Michaelis-Menten,

(1) $$v\;=\;\frac{V_{\max}\;\left[S\right]}{K_{m}+\left[S\right]}$$

where: *v*, is the initial velocity; *V*_max_, is the Maximum Velocity; [S], is the substrate concentration and *K*_m_, is the Michaelis-Menten Constant.

### 4.3. Inhibition Kinetic Characterization

To evaluate the time and concentration-dependence of the inhibition of urease activity, the initial rates of urea hydrolysis were measured in the standard reaction mixture in the presence of DSF (tetraethylthiuram disulphide), (C_2_H_5_)_2_N(C=S)S–S(S=C)N(C_2_H_5_)_2_, during different incubation periods. DSF was purchased from Sigma-Aldrich (Toluca, Mexico). In these experiments urease reactions were started by the addition of urea to a final concentration of 2 mM. The residual activity in percentage of the initial was plotted against the time of incubation and fitted to an exponential decay equation with a *plateau* (Equation (2)).

Equation (2): Enzyme inactivation,

(2) $$\mathrm{residual}\;\mathrm{activity}\;=\;\left(A_{0}-plateau\right)\left(e^{-kt}\right)+plateau,$$

where the residual activity is the observed activity upon the inhibition; *A*_0_, is the activity observed in the absence of the inhibitor; the *plateau*, is the value of Y minimum; *k*, is the observed one phase kinetic constant, and t is the time of incubation. Activity was transformed to percentage, where activity in the absence of DSF is 100%.

To evaluate the saturation of CVU with inhibitors, enzyme was incubated for one hour at 37 °C with different concentrations of inhibitors in the standard reaction mixture, the reactions were started by the addition of urea to a final concentration of 2 mM, followed by the measurement of the residual activity. Percentages of residual activity were plotted against inhibitor concentration. Since two types of behavior were observed, two different equations were used to fit the data. In one case, we observed a trend that produced a full loss of activity (Equation (3)), and in the other case a partial inhibition is observed, meaning that at enzyme saturation activity value is non-zero (Equation (4)).

Equation (3): Full-Inhibition Saturation Kinetics,

(3) $$\mathrm{residual}\;\mathrm{activity}\;\left(\%\right)\;=\;\frac{A_{0}\;IC_{50}^{h}}{IC_{50}^{h}+{\left[I\right]}^{h}}$$

where *h*, is the Hill coefficient; *IC*_50_ is the apparent inhibition constant; [I], is the inhibitor concentration. Residual activity and *A*_0_ is the same as in Equation (2).

Equation (4): Partially-Inhibition Saturation Kinetics,

(4) $$\mathrm{residual}\;\mathrm{activity}\;\left(\%\right)=\;\frac{A_{0}\;IC_{50}^{h}}{IC_{50}^{h}+{\left[I\right]}^{h}}+plateau$$

where parameters and variables are same as in Equation (3), with the difference that a *plateau* is included.

For the inhibition pattern urea saturation kinetics experiments were performed at fixed/variable concentrations of DSF and data were globally fitted to Equation (5).

Equation (5): Non-Competitive Inhibition Mechanism,

(5) $$v\;=\;\frac{\left(\frac{V_{\max}}{1+\frac{\left[I\right]}{K_{i}}}\right)\;\left[S\right]}{K_{m}+\left[S\right]}$$

where *K*_i_, is the Non-Competitive Inhibition Constant, and all other parameters and variables are previously defined.

Inhibition of CVU by Aldrithiol (2,2′-dipyridyl disulphide, here in after called DPS for dipyridine sulfide), captopril ((*S*)-1-(3-Mercapto-2-methyl-1-oxopropyl)-l-proline), bithionol (2,2′-Sulfanediylbis(4,6-dichlorophenol)) and quercetin were performed in a similar manner as for inhibition experiments with DSF. These inhibitors were purchased from Sigma-Aldrich (Toluca, Mexico). Inhibition kinetics data were fitted to the indicated equation. The protection of the activity by a given inhibitor (called I_2_), at a concentration equal to the *IC*_50_ value, over the inhibition produced by DPS was monitored measuring changes in initial velocity and plotting the percentage of inhibition for each treatment and expressed as mean ± SEM. Student t test was used to estimate statistical significance. Measurements of variance were performed using a one-way ANOVA. Differences were considered significant when *p* was less than 0.05. All experiments were performed at least three times, and in all cases the SEM justifies the accuracy of the values, except in the case of inhibition kinetics of bithionol and quercetin in which SEM is higher than 10% of the estimated value.

### 4.4. Binding Equilibrium Experiments

The binding of DSF was monitored after apparent equilibrium by measuring the intrinsic tryptophan fluorescence intensity changes at 37 °C using a spectrometer (Fluostar Omega, BMG). CVU was excited at 290 nm, and fluorescence intensity was recorded at 340 nm after an hour of incubation at 37 °C in the absence or presence of different concentrations of DSF. In order to discard solvent fluorescent changes, a control experiment was performed in which the different volumes of solvent (same as in the titration volumes used in ligand additions) were added to urease. Fluorescence intensity changes were plotted against the DSF concentrations and fitted to Equation (5).

Equation (6): Fluorescence binding saturation,

(6) $$\Delta IF\;=\;\frac{B_{\max}\;\left[\mathrm{DSF}\right]}{K_{d}+\left[\mathrm{DSF}\right]}$$

where *ΔIF*, is the change in fluorescence intensity at 340 nm; *B*_max_, is the maximum *ΔIF* and corresponds to the maximum binding; [DSF], is the disulfiram concentration and *K*_d_, is the dissociation constant.

### 4.5. DSF Docking

In order to test the possibility of DSF interacting with Cys592 of urease, docking experiments in JBU (PDB code: 3LA4) were performed by using AutoDock Vina and analyzing the 9 best scored solutions [35] with DSF and *N,N*-diethyldithiocarbamate (DTC). JBU three-dimensional structure was downloaded from PDB, and DSF was downloaded from Zinc Database (http://zinc.docking.org/substance/1529266). Three-dimensional structures where dock prepared using USCF-Chimera (https://www.cgl.ucsf.edu/chimera/), followed by the function Autodock Vina which was used to perform docking using a grid that corresponds to the whole tetrameric urease structure. The most abundant auto-validated complex model was analyzed, and the best model is presented.

## 5. Conclusions

Our kinetic experiments, docking models and comparative study of available urease sequences support the potential use of disulfiram to control urease activity. On the other hand, disulfiram and other compounds that contain thiol reactive groups can be used to redesign better and specific urease inhibitors. Here the docking approach seems to be adequate to predict whether compounds of this kind can interact with relevant Cys residues.

## Figures and Tables

**Table 1:** *IC*_50_ values of CVU inhibition by Sulphur-reactive-compounds and quercetin. Span represents the percentage of the inhibition, and ½ of the span was calculated from the difference between the *plateau* obtained by the fitting and 100% of the initial activity.

Compound	*IC*_50_ (µM)	*Plateau* (% of Initial)	½ of Span (% of Initial)
DSF	80.02 ± 1.30 × 10^−4^	0.00	50.00
Aldrithiol (DPS)	3.35 ± 0.81	0.00	50.00
Captopril	523.90 ± 39.62	0.00	50.00
Bithionol	376.50 ± 120.40	34.53 ± 18.23	67.26
Quercetin	154.70 ± 10.15	58.86 ± 1.52	79.43
